# Robotic Exoskeleton Gait Training in Stroke: An Electromyography-Based Evaluation

**DOI:** 10.3389/fnbot.2021.733738

**Published:** 2021-11-26

**Authors:** Valeria Longatelli, Alessandra Pedrocchi, Eleonora Guanziroli, Franco Molteni, Marta Gandolla

**Affiliations:** ^1^NearLab, Department of Electronics, Information and Bioengineering, Politecnico di Milano, Milan, Italy; ^2^Villa Beretta Rehabilitation Center, Valduce Hospital, Costa Masnaga, Italy; ^3^Department of Mechanical Engineering, Politecnico di Milano, Milan, Italy

**Keywords:** rehabilitation robotics, electromyography, coherence, exoskeletons, capacity score

## Abstract

The recovery of symmetric and efficient walking is one of the key goals of a rehabilitation program in patients with stroke. The use of overground exoskeletons alongside conventional gait training might help foster rhythmic muscle activation in the gait cycle toward a more efficient gait. About twenty-nine patients with subacute stroke have been recruited and underwent either conventional gait training or experimental training, including overground gait training using a wearable powered exoskeleton alongside conventional therapy. Before and after the rehabilitation treatment, we assessed: (i) gait functionality by means of clinical scales combined to obtain a Capacity Score, and (ii) gait neuromuscular lower limbs pattern using superficial EMG signals. Both groups improved their ability to walk in terms of functional gait, as detected by the Capacity Score. However, only the group treated with the robotic exoskeleton regained a controlled rhythmic neuromuscular pattern in the proximal lower limb muscles, as observed by the muscular activation analysis. Coherence analysis suggested that the control group (CG) improvement was mediated mainly by spinal cord control, while experimental group improvements were mediated by cortical-driven control. In subacute stroke patients, we hypothesize that exoskeleton multijoint powered fine control overground gait training, alongside conventional care, may lead to a more fine-tuned and efficient gait pattern.

## 1. Introduction

Stroke is the leading cause of chronic disability in adults (Gorelick, 2019). Stroke survivors often present deficits in mobility, balance, and coordination, drastically limiting their activities of daily living (ADLs) and social interaction (Rössler et al., 2020). The recovery of independent ambulation is without any doubt one of the key ADLs to be targeted with the rehabilitation program. From a functional perspective, it has been largely demonstrated that important ingredients for a successful neurorehabilitation program might be identified in early intervention, high dose, high intensity, and task-specific practice. Indeed, during the acute stages of post-stroke recovery, repetitive, high dose, task-specific training has been found to enhance beneficial neuroplasticity that may accelerate functional recovery and restore healthy gait after stroke (Kwakkel et al., 2004; Langhorne et al., 2011; Maier et al., 2019). With a particular focus on gait, it has been demonstrated that a particular effect can be obtained by increasing the number of repetitions, characterized by a kinematic close to a physiological pattern able to induce a proper inter-joint and inter-limb coordination (Esquenazi and Talaty, 2019).

Current conventional therapy can produce remarkable improvements in ambulation and motor functions. However, given the increasing population needing rehabilitation assistance and lack of time and human resources, physical therapists may not always be able to provide enough high dose, task-specific repetitive gait training during the acute stages of recovery, where maximum physical assistance is required (Louie and Eng, 2016). In this context, recent researchers developed technologies to support the work of physical therapists and to be mainly focused on delivering a high number of gait repetitions in the post-acute phase. In the attempt to early verticalize patients, devices like body-weight supported treadmill were developed to increase the delivered dose. However, these devices showed limited evidence of efficacy (Nilsson et al., 2001; Mehrholz et al., 2017). This could be due to the lack of the task-oriented bit, which is to cover an even short distance in the case of walking (Nolan et al., 2020).

Technological advancements focused on the development of powered wearable robotic exoskeletons, electromechanical devices with bilateral motorized assistance at the knee and hip joints in the sagittal plane, including commercial devices such as Ekso (https://eksobionics.com), Re-Walk (https://rewalk.com/), and Indego (www.indego.com). In addition to body-weight supported treadmills, exoskeletons may be used in the early phase after injury. They can provide tunable high intensity and high dose rehabilitation sessions with effective over-ground ambulation, reducing the effort of the therapist. Indeed, the therapists may focus on training cues and feedback to drive gait quality in a stabilized system that is provides upright support to the trunk and lower limb.

When comparing conventional therapy (control group) and exoskeleton-based therapy (experimental group), clinical trials have demonstrated that the experimental group showed comparable or slightly increased improvements, mainly using clinical evaluation or tests (Peurala et al., 2009; van Nunen et al., 2015; Cho et al., 2018; Molteni et al., 2018; Nolan et al., 2020). This may support the use of such technologies in the standard rehabilitation pathway. However, such a technology should be delivered to specific patients, and little is known about the underlying mechanisms that drive robotic-mediated neural plasticity during the rehabilitation process. Indeed, a deep understanding of the underlying motor relearning mechanisms may contribute toward effective patient stratification in rehabilitation treatments. Since MRI, functional MRI, EEG, and similar solutions are difficult to be routinely performed in clinical settings, surface electromyography (sEMG) may be an option for an indirect investigation of neural plasticity. Despite its potential demonstrated in the literature, sEMG has been demonstrated to have several limitations toward effective clinical application (Campanini et al., 2020; Goffredo et al., 2020). Indeed, the sEMG signal is influenced by several factors, including inter-electrodes distance, skin-electrode impedance, or electrode placement with respect to underlying muscle fibers, which surface electromyography for the non-invasive assessment of muscles (SENIAM) directions, have however, well addressed. Most of the current limitations are for effective and meaningful signal processing. Indeed, signal pre-preprocessing is well-defined and quite common toward different applications to obtain an sEMG envelope. Still, difficulties within the community may be identified in reliable real-time muscle activation onset and movement identification procedure (e.g., steps identification). Moreover, repeatability and signal normalization between different sessions have limited the use of sEMG as the primary outcome measure. Despite all these reasons, if carefully acquired and analyzed, sEMG signals have been demonstrated to be a key-hole to human nervous system plasticity (Kitatani et al., 2016).

The present study investigates the gait rehabilitation process and evaluates motor re-learning in patients with subacute post-stroke by analyzing and comparing lower limb muscular activation patterns. Two different rehabilitation strategies were considered: (i) conventional physical therapy, and (ii) exoskeleton-assisted rehabilitation, in addition to standard therapy sessions. A quantitative index that summarizes the clinical outcomes of patients was computed to compare the clinical assessments and the results obtained from the muscular activity analysis. Three gait performance indexes [i.e., Gait Metric (GM) Ricamato and Hidler, 2005, Burst Duration Similarity Index (BDSI) Androwis et al., 2018, and agonist-antagonist coherence function Kitatani et al., 2016] were extracted from patients' surface EMG signals of lower limbs before and at the end of the treatment, to compare changes in muscular activation resulting from the two different rehabilitation therapies. In particular, GM (Ricamato and Hidler, 2005) and BDSI (Androwis et al., 2018) were used to analyze muscular activation during gait in terms of amplitude and timing. The investigation of agonist-antagonist muscles coherence (Kitatani et al., 2016) allowed a preliminary evaluation of changes in corticospinal neural drives consequent to stroke, and possible neural plasticity promoted by rehabilitation.

## 2. Methods

A single-blinded pilot study was conducted at the Villa Beretta Rehabilitation Center (Costa Masnaga, LC, Italy). The study was approved by the ethical committee of the rehabilitation center (Comitato Etico Interaziendale delle Province di Lecco, Como e Sondrio, Prot. 48892/15 23/11/2015). All subjects gave informed written consent in accordance with the Declaration of Helsinki. Patients were recruited between March 2016 and March 2018. The present study is part of a multicenter, parallel study about the effects of exoskeleton-assisted training on patients with post-stroke, whose details can be found in Goffredo et al. (2019a).

### 2.1. Participants

This study involved both patients with post-stroke and healthy subjects. Considering patients with post-stroke, inclusion criteria were as follows: (i) the first-ever stroke affecting the lower limbs; (ii) level of spasticity at the hip, knee, and ankle levels <3, as detected by Modified Ashworth Scale (Blackburn et al., 2002); (iii) stroke event happened from 2 weeks up to 6 months post the acute event (i.e., subacute patients); (iv) age between 18 and 80 years; (v) ability to fit into the exoskeleton; (vi) no significant limitation of hip and knee joints range of motion; (vii) ability to tolerate upright standing for at least 60 s without loss of pressure due to verticalization; (viii) sufficient upper extremity strength and balance for walking with the device; and (ix) ability to give written consent and comply with the study procedures. Moreover, a group composed of healthy volunteers with no neurological or orthopedic impairment was recruited to derive the reference physiological gait pattern.

### 2.2. Experimental Set-Up

This study involved the overground wearable powered exoskeleton Ekso GTA^TM^, Ekso Bionics, USA. Ekso is a wearable bionic suit, which enables individuals with lower limb disabilities and minimal forearm strength to stand, sit, and walk over a flat hard surface under the supervision of a therapist. It is equipped with four battery-powered motors at the hips and knees. Considering the type of actuation, in the present study, ProStepPlus and Bilateral Max Assist were used as default settings. Therefore, each step of the patient was triggered by the lateral weight shift by the user, and Ekso provided full power to both legs. No strength was required from the patient to achieve walking, but only proper balance and weight shifts. Ekso was adjusted to fit the anthropometric data of patients. Before starting the training period, a physiotherapist checked for correct alignment of the joints of the subject with the robot and areas of increased skin pressure, adjusting the fit accordingly.

The gait cycle kinematic parameters of the exoskeleton were fine-tuned using sEMG to define a customized and tailored robotic treatment, as proposed in Gandolla et al. (2018). Bilateral muscle activity was recorded during overground walking with the FREEEMG wireless electromyograph (BTS Bioengineering, Garbagnate Milanese, Milano, Italy). In particular, we bilaterally recorded the Tibialis Anterior (TA), Soleus (SOL), Rectus Femoris (RF), and Semitendinosus (ST). We chose a couple of agonist and antagonist muscles in the distal and proximal compartment, respectively. In particular, in the distal compartment, we recorded the SOL because it is the only calf muscle mono-joint, and its action is performed at ankle level, and the TA Anterior, since it is the principal ankle flexor, and it is important for the foot clearance.

### 2.3. Functional Outcomes

A functional assessment has been performed before (i.e., T0) and after the treatment (i.e., T1) with multiple clinical scales combined into a comprehensive index called Capacity Score, as described in Gandolla et al. (2015). This comprehensive index allowed to evaluate the overall impact of the rehabilitation processes on the motor capabilities of the subjects and their abilities to perform everyday life activities. In particular, six of the most significant clinical scales for gait assessment were included: the 5-item modified Barthel Index (Hobart and Thompson, 2001), the Motricity Index (Cameron and Bohannon, 2000), the 10 meters walk test (Tyson and Connell, 2009), the 6 min walk test (Tyson and Connell, 2009), the Functional Ambulation Category (Mehrholz et al., 2007), and the Trunk Control Test (Duarte et al., 2002). For what concerned the 5-item Barthel Index, the Motricity Index, the Functional Ambulation Category, and the Trunk Control Test, mean values for healthy subjects were set equal to the maximum score achievable during the test. For the 6 min walk test and the 10 meter walk test, mean values referred to a healthy population were derived from literature (Steffen et al., 2002; Bohannon and Andrews, 2011). Minimum Detectable Change (MDC) for the Functional Ambulation Category and the Trunk Control Test were set as the minimum difference between two consecutive scores according to the structure of each test. MDC for the 6 min walk test and the 10 meter walk test were, respectively, equal to 60.98 m and 0.11 m/s (Perera et al., 2006). According to literature, the MDC in the score of the 5-item Barthel Index was 7.84 (Park, 2018), while for the Motricity Index it was 12.92 (Fayazi et al., 2012). All outcomes were equally weighted to compute the final score. The higher the Capacity Score is, the higher the functional capacities of the patient are. Therefore, the difference between the Capacity Score at T1 and T0 assesses the functional improvement.

### 2.4. Instrumental Outcomes

#### 2.4.1. Muscular Activity Assessment and Steps Segmentation

Electromyography signals were acquired with a frequency of 1 kHz. A standard pre-processing (Solnik et al., 2008) was applied to the sEMG signals for all included participants, including high-pass filtering with a 3rd order Butterworth filter at 20 Hz, rectification, and low-pass filtering with a 3rd order Butterworth filter at 4 Hz. The step segmentation was obtained from EMG signals. We implemented a moving window algorithm with an adaptive threshold, as detailed in Gandolla et al. (2018). The algorithm was used to identify the deactivation of SOL muscle, which was then used to trigger the step segmentation. The algorithm was based on the monophasic activation of SOL during the physiologic gait cycle. Since this property was also proved reliable in the non-paretic leg of patients with stroke (Gandolla et al., 2018), a non-paretic SOL activation profile was used to segment both patients' steps (i.e., paretic and non-paretic limbs sEMG signals). For the healthy subjects, the right SOL deactivation was arbitrarily selected to segment steps. The accuracy of the step identification technique was evaluated by comparing the number of steps obtained by the algorithm with that coming from footswitches placed under the shoes of the subjects. This signal was available for all healthy volunteers and only for a reduced number of patients.

For each subject, EMG signals from the segmented single steps were rescaled in time to obtain the same duration of the mean step duration of the healthy group. Then, data were averaged in amplitude and normalized to their own maximum amplitude. This pre-processing phase allowed to obtain for each muscle of each subject a muscular activation profile normalized in amplitude between 0 and 1 and normalized in time between 0 and 100% of the gait cycle. As a consequence of this segmentation algorithm, the obtained step cycle for the non-paretic limb started from the toe-off phase, and the step cycle of the paretic limb started from its loading response phase. Thus, normative physiologic activation profiles of the analyzed muscles were obtained by average within the muscular profiles of seven healthy subjects. Through qualitative inspection of the morphology of the muscle activation profiles in healthy subjects and taking into account muscular dynamics reported in the literature define, the 40% of amplitude peak was chosen to define a threshold to obtain the activation and deactivation phases of each muscle during a single step cycle.

#### 2.4.2. Gait Metric

The GM compares the dynamic properties (i.e., amplitude and timing) of locomotor EMG patterns to normative gait-related EMG patterns generated under comparable walking conditions (Ricamato and Hidler, 2005). In particular, the GM index provides a quantitative measure of the EMG pattern by calculating two parameters: (i) the magnitude of the area of normative activation and (ii) the phase shift with respect to the normative gait-related EMG pattern. Specifically, the metric rewards EMG activity when the muscle is active (i.e., greater than the set threshold) in the portion of the gait cycle where it is physiologically active, and also when EMG activity is inactive (i.e., below the threshold) when the muscle is in the portion of the gait cycle where it is normally inactive. In the case of the magnitude component, the metric also penalizes the opposite conditions corresponding to the situation where the muscle is active when it should be silent and vice-versa. The normative activation was obtained by thresholding normative EMG profiles, as explained in section 2.4.1. The GM index was computed as described in the literature (Ricamato and Hidler, 2005).

#### 2.4.3. BDSI

The BDSI quantifies the similarity between two muscles activations timing by measuring co-activation (i.e., common active regions between the patient and the normative profile) and co-deactivation (i.e., common inactive regions between the patient and the normative profile) during the gait cycle (Androwis et al., 2018). Once active/inactive regions referred to single step cycle have been defined for each muscle as in section 2.4.1, we quantified the match between the two EMG signals. The BDSI was calculated as Equation (1):


(1) $$\begin{array}{l} BDSI=\frac{sum\left(ON\text{\_}timing\right)+sum\left(OFF\text{\_}timing\right)}{N}*100 \end{array}$$

where BDSI is the computed index, *ON*_*timing* was a binary vector of length N with 1 indicating simultaneous activation of *s*_1_ and *s*_2_ and 0 otherwise; *OFF*_*timing* was a binary vector of length N with 1 indicating simultaneous inactivation of *s*_1_ and *s*_2_ and 0 otherwise.

#### 2.4.4. EMG-EMG Coherence

We conducted a coherence analysis of paired surface EMG recordings to quantitatively evaluate the descending neural drive from the Sensory-Motor Cortex during gait (Halliday et al., 2003). In particular, we computed the coherence between agonist and antagonist muscles, taking into account a couple of proximal muscles (RF-ST) and a couple of distal muscles (TA-SOL). To perform coherence extraction, it is necessary to set a fixed-length window in which the two agonist-antagonist muscles are expected to be both active (Halliday et al., 2003). According to normative muscular activation patterns described in the literature (Winter and Yack, 1987; Gandolla et al., 2018), a 200 ms slot was selected in the initial part of the mid-stance phase for the TA-SOL couple, while an epoch belonging to the terminal swing phase was chosen for the RF-ST couple. The coherence was computed as described in Kitatani et al. (2016). The coherence function was evaluated in three frequency bands: (i) 5–10 Hz (i.e., alpha band), (ii) 15–30 Hz (i.e., beta band), and (iii) 30–45 Hz (i.e., gamma band). According to the literature (Halliday et al., 2003; Kitatani et al., 2016), healthy subjects have coherence between agonist-antagonist muscles in the alpha band but not in beta and gamma bands. Conversely, in patients with post-stroke, agonist-antagonist muscle coherence has been shown to be reduced in the alpha band, but it is present in beta and gamma bands. The alpha band has been shown to be related to the spinal drive, while the beta and gamma bands to cortical activity (Kitatani et al., 2016). To quantitatively evaluate the magnitudes of the coherence, we computed the magnitude-squared coherence and calculated the area under the coherence curve within the three frequency bands. Moreover, coherence was considered significant if higher than the 95% confidence limit under the hypothesis of independence, calculated as Equation (2) (Halliday et al., 1995):


(2) $$\begin{array}{l} ConfidenceLimit_{95\%}=1-{\left(0.05\right)}^{\frac{1}{L-1}} \end{array}$$

where *L* was the number of steps. The same number of steps was used to compare coherence results from the same subject between T0 and T1.

### 2.5. Experimental Protocol

In this study, enrolled patients were divided into two groups: i) CG, which performed conventional rehabilitation therapy, and ii) EG, which underwent exoskeleton-assisted gait rehabilitation in addition to conventional therapy. Both groups underwent five training sessions per week, for 4 weeks resulting in 20 rehabilitation sessions. For the EG, the number of exoskeleton-assisted rehabilitation sessions was 12 over 20 sessions, and it was provided three times per week. During the other eight sessions, the EG underwent conventional therapy as the CG. The duration of every single session was 60 min, including the exoskeleton donning and doffing. Ekso fine-tuning was defined during the first evaluation session, and did not change during the 12 trial sessions. It included the setting of the three main setting parameters: (i) lateral shift (displacement of body weight under the foot of the patient), (ii) swing time, and (iii) step length. We used ProStep Plus Ekso setting program, through which steps are triggered by the lateral weight shift of the user, and Bilateral Max Assist, in which Ekso provides full power to both legs and no strength is required. Starting from this configuration, Ekso parameters were chosen using sEMG (Gandolla et al., 2018). The definition started with the first parameter to be set (i.e., lateral shift). A series of overground gait trials were performed, while setting the parameter to different values. The gait trials were minimum of three, where the default value, and higher and lower settings were tested. By observing the gait quality and sEMG signals acquired during walking, the expert Ekso therapist and the rehabilitation team selected the best parameter setting. The first parameter was then fixed, and the next parameters were considered in a recursive procedure until Ekso was properly set.

Clinical outcomes and sEMG signals were acquired before (i.e., T0) and after the treatment (i.e., T1). sEMG signals were recorded during a single trial of overground walking performed by patients indoors, along a long, flat straight enclosed corridor with a hard surface. During clinical and instrumental measurements, patients from the EG were not wearing the exoskeleton. When coming to healthy subjects, sEMG signals were recorded during five consecutive autonomous walking trials in the same environmental situation. During the recording, healthy subjects were not wearing the exoskeleton.

### 2.6. Statistical Analysis

Mann-Whitney *U*-test for independent samples for continuous outcome measures and Pearson chi-squared test for frequencies were used to compare the characteristics of participants at baseline between the two groups. For Capacity Score, GM index and BDSI, generalized linear mixed-model analyses were performed, with group and time entered as fixed effects, time by the group as the interaction term, age, time since stroke and Motricity Index at T0 as covariates, patients as a random factor, and the outcome measurement (i.e., Capacity Score, GM index, and BDSI) as a dependent variable. A *post-hoc* analysis considering the groups independently was performed to estimate T1-T0 changes in case of a significant time effect. In case of significant group or interaction effects, the between-group changes at T1 were also estimated, with age, time since stroke, and Motricity Index at T0 as covariates. We conducted these analyses for each muscle of both paretic and non-paretic leg and the couples of proximal (i.e., RF-ST), distal (i.e., TA-SOL), agonist (i.e., RF-SOL), and antagonist (i.e., ST-TA) muscles. For the coherence analysis, the Wilcoxon test for paired samples was used to compare the area under the curve between T1 and T0. Percentages of clinically improved patients at T1 were finally computed. Specifically, the clinical improvement related to coherence was considered significant if the patient had no significant coherence at T0 and significant coherence at T1 for the alpha band, and vice-versa for the beta and gamma bands. The Pearson chi-squared test was used to compare percentages of improved subjects between groups. The statistical analyses were performed using SPSS v27.

## 3. Results

### 3.1. Participants

About 29 patients with subacute stroke were recruited. The CG and EG were composed of 14 and 15 patients, respectively. Table 1 shows the group comparison at the baseline (i.e., T0). No significant differences have been observed. The median time since stroke was 34.50 days [28–51] for the CG and 40 [30–64] days for the EG. Both groups were unbalanced, with a higher percentage of men, ischemic stroke, and the most frequently affected size was the left one. However, we did not detect between groups unbalances (*p* > 0.05). Considering clinical scales at T0, we can observe greater scores for the CG than the EG. Nevertheless, they were not significantly different (*p* > 0.05). The healthy group was composed of seven healthy subjects, with a median age of 30 [23–32] years, and it included four men and three women.

**Table 1:** Demographic characteristics and functional outcome measures at baseline (*N* = 29).

	**Control group** **(***N*** = 14)**	**Experimental group** **(***N*** = 15)**	***P*** **-value**
Age^*a*^	68 [64–70]	65 [52.25–73.75]	0.813^b^
Time since stroke (days)	34.50 [28–51]	40 [30 –64]	0.451^c^
Sex (Male/Female)	11/3	10/5	0.474^c^
Affected size (Left/Right)	8/6	9/6	0.876^c^
Etiology (Hemorrhagic/Ischemic)	2/12	5/10	0.231^c^
Motricity index (0–100)^a^	55 [26.50–68.50]	29 [18.75–43.13]	0.085^b^
5-item barthel index (0–55)^a^	19 [9–25]	12 [9–14]	0.164^b^
10 meters walk test (m/s)^a^	0.22 [0.11–0.59]	0.17 [0.12–0.22]	0.603^b^
6 min walking test (m)^a^	40 [6–150]	29 [15–49]	0.571^b^
Functional ambulation category (0–5)^a^	1 [0–3]	0.50 [0 – 1]	0.094^*b*^
Trunk control test (0–100)^a^	49 [37–75]	43 [36–61]	0.285^b^

a *Median [I–III Quartile]*;

b *Mann-Whitney U-test*;

c *Pearson chi-squared test*.

### 3.2. Functional Outcomes Results

The Table 2 shows the results of the Capacity Score analysis. We detected a significant time effect (*p* < 0.001), while we did not observe significant group nor interaction effects. The *post-hoc* analysis confirmed that both groups improved at T1 (*p* < 0.001) and that a between-group difference was not present (*p* = 0.350). These results confirmed those obtained in the clinical study of Goffredo et al. (2019a), which observed that robotic gait training can produce significant improvement in clinical outcomes a the conventional therapy.

**Table 2:** Changes over time and between group for Capacity Score (*N* = 29).

**Outcome**	**Group**	**T0^a^**	**T1^a^**	***P*** **-value^b^**	***P*** **-value^b^**	***P*** **-value^b^**	**T1–T0**		**Group change at T1**	
				**(Time)**	**(Group)**	(Time ^*^ Group)	**Change (SE)^c^**	***P*** **-value^d^**	**Change (SE)^c^**	***P*** **-value^e^**
Capacity Score	CG	5.06 [3.63–9.63]	9.06 [6.97–13.83]	**<0.001**	0.326	0.459	3.547 (0.331)	**<0.001**	–1.070	0.350
	EG	3.99 [3.29–5.50]	6.63 [5.76–7.61]				3.052 (0.273)	**<0.001**	(1.122)	

*Significant differences (p-value < 0.05) are reported in bold*.

*T0, Assessment before the intervention; T1, Assessment after the intervention; CG, control group; EG, experimental group*.

a *Median [I-III Quartile]*;

b *Generalized linear mixed model*;

c *Mean difference (SE)*;

d *Generalized linear mixed model on each group separately*;

e *Generalized linear mixed model to compare groups at T1*.

### 3.3. Instrumental Outcomes Results

**Figure 1:**
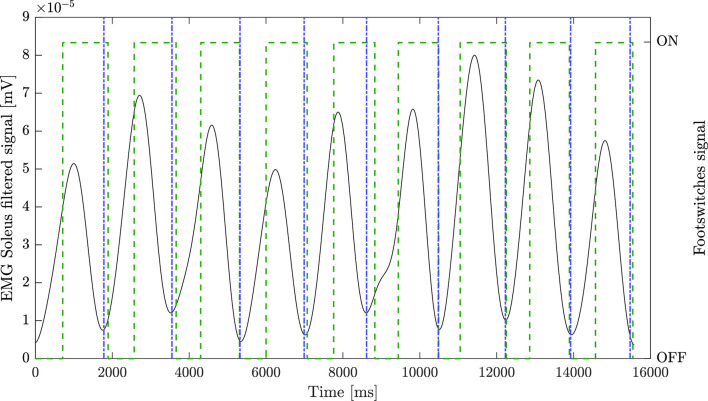
Step segmentation algorithm results. Black line represents the pre-processed soleus (SOL) signal of the non-paretic leg, green dashed line represents footswitch signal placed under the shoe of the patient, blue dash-dot line represents EMG-based step segmentation.

The step segmentation algorithm was characterized by an accuracy of 100% for the healthy group. Considering patients, instead, the footswitches signal were available for 21 sessions. The median accuracy was 100% [93.75–100%]. The algorithm correctly identified all steps in 12 recordings. The maximum error was equal to three steps, and it occurred in one patient. Figure 1 reports an example of the segmentation algorithm results together with the corresponding signal from the footswitches on the SOL filtered muscle.

The EMG analysis on the healthy CG was conducted to derive normative reference profiles and indices. The normative results for the GM index were: 0.73 ± 0.10 for the TA, 0.83 ± 0.03 for the SOL, 0.66 ± 0.08 for the RF, and 0.74 ± 0.05 for the SM. For the BDSI, we obtained the following mean results: 80.22 ± 12.11 for the TA, 92.38 ± 5.55 for the SOL, 68.13 ± 6.79 for the RF, and 81.89 ± 11.01 for the SM.

**Figure 2:**
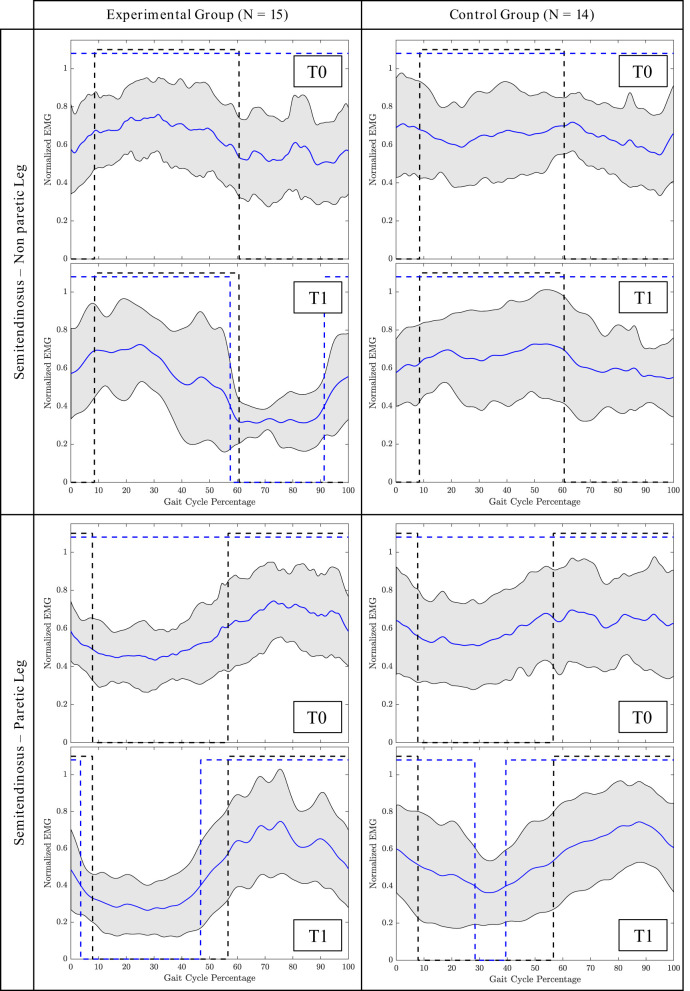
Mean gait cycle of the Semitendinosus (ST) muscle before the intervention (T0) and after the intervention (T1). Blue line = Mean patients' activation; Grey area = Patients' SD; Black dashed line = Normative activation profile; Blue dashed line = Patients' activation profile (active/non-active muscle window). Profile are represented as a function of the gait cycle. In the non-paretic leg, the gait cycle starts from the toe off, in the paretic leg, it starts from the loading response phase.

The average profile of the ST muscle of both groups of non-paretic and paretic legs before and after the intervention is shown in Figure 2.

The results of the GM index for the patients are reported in Table 3. We observed a significant time effect for the SOL of the non paretic leg (*p* = 0.035) and the couple ST-TA (p-value = 0.015). The *post-hoc* analysis demonstrated that the improvement between T0 and T1 was significant only for the EG in both cases (*p* = 0.034 for SOL, *p* = 0.018 for ST-TA). A significant group effect was detected at T1 for the non-paretic ST and the non-paretic proximal muscles (ST-RF). Finally, we obtained a significant interaction effect for the ST muscle and the paretic leg proximal muscles. A between-group change of 0.094 (*p* = 0.025), and 0.057 (*p* = 0.043) in favor of the EG was found at T1 for the paretic ST muscle and paretic ST-RF, respectively.

**Table 3:** Changes over time and between group for gait metric (*N* = 29).

**Outcome**	**Group**	**T0^a^**	**T1^a^**	***P*** **-value^b^**	***P*** **-value^b^**	***P*** **-value^b^**	**T1–T0**		**Group change at T1**	
				**(Time)**	**(Group)**	(Time ^*^ Group)	**Change (SE)** ^c^	***p*** **-value** ^d^	**Change (SE)** ^c^	***p*** **-value** ^e^
SOL non paretic	CG	0.59 [0.50–0.65]	0.64 [0.53–0.67]	**0.035**	0.861	0.389	0.028 (0.031)	0.373	−−	−−
	EG	0.52 [0.47–0.58]	0.57 [0.53–0.65]				0.065 (0.30)	**0.034**		
ST non paretic	CG	0.40 [0.36–0.44]	0.46 [0.39–0.51]	0.080	**0.017**	0.607	−−	−−	0.094	**0.025**
	EG	0.46 [0.39–0.53]	0.54 [0.42-0.61]						(0.039)	
ST paretic	CG	0.44 [0.40–0.69]	0.42 [0.40-0.47]	0.845	0.086	**0.041**	−−	−−	0.129	**0.007**
	EG	0.41 [0.40–0.54]	0.50 [0.44–0.56]						(0.043)	
Proximal non paretic	CG	0.45 [0.43–0.50]	0.47 [0.44–0.53]	0.528	**0.008**	0.647	−−	−−	0.057	**0.035**
	EG	0.48 [0.45–0.54]	0.50 [0.46–0.60]						(0.026)	
Proximal paretic	CG	0.47 [0.44–0.63]	0.48 [0.44–0.51]	0.932	0.455	**0.048**	−−	−−	0.057	**0.043**
	EG	0.46 [0.43–0.52]	0.50 [0.45-0.55]						(0.027)	
Antagonists non paretic	CG	0.49 [0.45–0.52]	0.53 [0.50–0.55]	**0.015**	0.190	0.380	0.028 (0.024)	0.260	−−	−−
	EG	0.47 [0.46-0.55]	0.55 [0.47–0.61]				0.058 (0.024)	**0.018**		

*Significant differences (p-value < 0.05) are reported in bold*.

*T0, Assessment before the intervention; T1, Assessment after the intervention; CG, control group; EG, experimental group*.

a *Median [I-III Quartile]*;

b *Generalized linear mixed model*;

c *Mean difference (SE)*;

d *Generalized linear mixed model on each group separately*;

e *Generalized linear mixed model to compare groups at T1*.

The results of the BDSI confirmed what we observed for the GM index, except the non-paretic SOL muscle (Table 4). Indeed, we obtained a significant time effect for the non-paretic ST (*p* = 0.033) and the couple of non-paretic ST-TA (*p* = 0.009). The *post-hoc* analysis confirmed that the time effect was present only in the EG. For the non-paretic ST, as well as for the non-paretic proximal muscles couple, we also detected a significant group effect. A significant interaction effect was revealed for the paretic ST (*p* = 0.039) and for the paretic ST-RF couple (*p* = 0.042). At T1, we observed greater changes in favor of the EG for the following comparisons: non-paretic ST (*p* = 0.041), paretic ST (*p* = 0.014), non paretic proximal muscles (*p* = 0.034), and paretic proximal muscles (*p* = 0.013).

**Table 4:** Changes over time and between group for burst duration similarity index (*N* = 29).

**Outcome**	**Group**	**T0^a^**	**T1^a^**	***P*** **-value^b^**	***P*** **-value^b^**	***P*** **-value^b^**	**T1–T0**		**Group change at T1**	
				**(Time)**	**(Group)**	(Time ^*^ Group)	**Change (SE)** ^c^	***p*** **-value** ^d^	**Change (SE)** ^c^	***p*** **-value** ^e^
ST non paretic	CG	36.08 [36.08–38.08]	43.43 [30.64–60.73]	**0.033**	**0.027**	0.741	5.293 (3.404)	0.134	9.929	**0.041**
	EG	36.08 [36.08–52.47]	46.66 [38.55–65.61]				7.190 (4.500)	**0.023**	(5.800)	
ST paretic	CG	39.81 [37.81–71.15]	39.18 [38.10–46.27]	0.761	0.155	**0.039**	−−	−−	15.236	**0.014**
	EG	40.81 [33.83–53.75]	52.18 [41.00–60.85]						(5.729)	
Proximal non paretic	CG	42.96 [42.96–47.74]	45.78 [41.68–54.98]	0.476	**0.009**	0.745	−−	−−	6.892	**0.034**
	EG	46.31 [45.18–53.81]	50.39 [43.76–60.07]						(3.876)	
Proximal paretic	CG	47.53 [43.43–68.86]	46.58 [43.43–51.56]	0.790	0.878	**0.042**	−−	−−	5.537	**0.013**
	EG	43.43 [42.15–50.40]	49.96 [43.43–56.59]						(3.363)	
Antagonists non paretic	CG	45.98 [41.72–51.91]	54.84 [48.76–58.09]	**0.009**	0.278	0.465	4.560 (3.004)	0.143	−−	**−−**
	EG	44.48 [41.74–56.07]	57.93 [46.45–64.69]				7.921 (3.410)	**0.029**		

*Significant differences (p-value < 0.05) are reported in bold*.

*T0, Assessment before the intervention; T1, Assessment after the intervention; CG, control group; EG, experimental group*.

a *Median [I-III Quartile]*;

b *Generalized linear mixed model*;

c *Mean difference (SE)*;

d *Generalized linear mixed model on each group separately*;

e *Generalized linear mixed model to compare groups at T1*.

**Figure 3:**
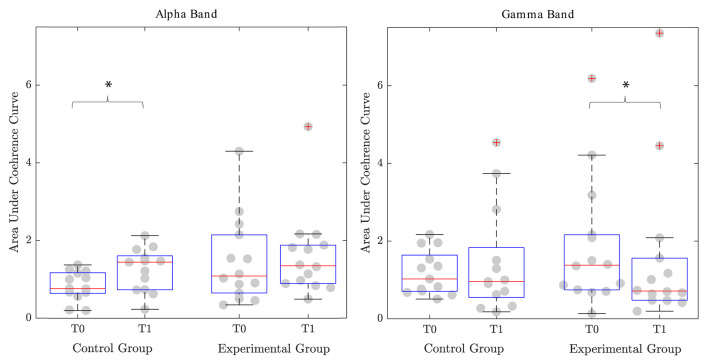
Area under the coherence curve for the couple of proximal agonist-antagonist muscles (Rectus Femoris-Semitendinosus). Significant differences (*p*-value <0.05) are reported with ^*^. T0, Assessment before the intervention; T1, Assessment after the intervention.

Considering the coherence, it was not possible to compute it for each trial. Indeed, some participants did not show a slot in the correct phase of the gait cycle where the agonist and the antagonist muscles are supposed to be simultaneously active. We considered only patients for whom it was possible to define the coherence both at T0 and T1. Specifically, for the EG, we were able to compute the coherence for 11 participants for the non-paretic and the paretic TA-SOL couples, 14 subjects for the non-paretic RF-ST couple, and nine participants for the paretic RF-ST muscles. Instead, in the CG, we successfully calculated the coherence for 9, 13, 11, and 10 patients for the non-paretic TA-SOL, non-paretic RF-ST, paretic TA-SOL, and paretic RF-ST couples, respectively. We firstly compared the area under the coherence curve with the Wilcoxon test. We obtained significant improvement in favor of the CG in the alpha band for the RF-ST couple of the non-paretic leg (*p* = 0.041). The median area increased from 0.76 [0.64–1.17] to 1.44 [0.73–1.61]. In the EG, instead, we observed a slight improvement from 1.03 [0.63–2.14] to 1.34 [0.89–1.88]. However, this change was not significant (*p* = 0.397). For the beta band, we did not obtain significant changes. For the gamma band, in the EG, the area underlying the coherence curve physiologically decreased in the RF-ST couple of the non-paretic leg (*p* = 0.017). Indeed, at T0 the area was 1.36 [0.73–2.16], while it decreased to 0.70 [0.47–1.56] after the intervention. In the CG, instead, the area decreased from 1.02 [0.70–1.63] to 0.96 [0.54–1.83], but it was not a significant change (*p* = 0.929) (Figure 3). The Pearson Chi-squared test further confirmed this result. Indeed, the proportion of clinically improved patients was 85.71% for the EG and 7.69% for the CG. This difference was significant (*p* < 0.001).

## 4. Discussions

One of the key goals of lower limb rehabilitation after stroke is the recovery of the symmetric and efficient gait pattern. The physiological gait is indeed the reference for a functional yet metabolically and dynamically fine-tuned lower limb motor control to restore a biomechanical efficient gait. The gait pattern of healthy individuals is characterized by a rhythmic and coordinated activation of lower limb muscles to ensure proper interlimb/joints coordination. After the rehabilitation process, patients with post-stroke usually recover their walking capacity, but the gait pattern often shows compensatory strategies that produce an inefficient gait pattern due to non-rhythmic and asymmetric gait, with relevant ipsilesional limb compensations for the paretic one dysfunctions. In this study, we identify the rhythmic sequence of muscular activation within the gait cycle as detected by EMG signals as an index of the similarity between the patients' and the physiological gait. The rehabilitation should pursue a double aim: (i) it should induce a functional gain (quantifiable in terms of velocity, endurance, safety, etc.), and (ii) it should guide the patient toward the recovery of rhythmic efficient gait control (quantifiable in terms of muscles activation pattern within the gait cycle). In the context of neurorehabilitation, the integration of wearable powered robotic exoskeletons, that allows a fine-tuning of gait parameters during overground training, with conventional physiotherapy could be essential during the subacute phase of stroke rehabilitation, when plasticity is high, to maximize functional positive adaptive plasticity of motor control, avoid maladaptive plasticity, and enhance training benefits and long-term outcomes (Androwis and Nolan, 2017). Conventional trials in this field assess relearning, usually reporting results on standard clinical scales, i.e., they usually report only on functional gain. However, they do not provide information on gait efficiency or the central and corticospinal pathways.

This study evaluated the impact of wearable powered robotic (Ekso Bionics) overground gait therapy compared to conventional care alone on patients with subacute post-stroke. In particular, we focused on the timing, amplitude, and frequency analysis (i.e., coherence) of the EMG profiles of four key muscles: TA, SOL, RF, and ST. Together with the instrumental outcomes, we computed the Capacity Score, a comprehensive index that summarizes six clinical scales related to lower limb motor abilities and functionality during daily life.

Both treatment and CG improved from a functional perspective (i.e., Capacity Score results). This is in line with previous studies on robotic-assisted rehabilitation (Mazzoleni et al., 2017; Bruni et al., 2018; Goffredo et al., 2019b; Taki et al., 2020).

However, the strategies used by the two types of treatments are different. Conventional therapy primarily aims at the recovery of the driven strategy of a patient. In other words, the therapy included muscle strengthening exercises and stretching of the lower limb, and static and dynamic exercises for the recovery of balance in the supine and standing positions using assistive devices; training gait exercises with parallel bars or in open spaces performed both with and without assistive devices; training to climb up and down stairs; exercises to improve proprioception in the supine, sitting and standing positions, using a proprioceptive footboard; exercises to improve trunk control to recover overground gait according to the self-driven strategy of the patient.

Overground wearable powered exoskeleton-assisted rehabilitation as well aims at functional recovery, but it guides lower limb joints (and, in particular, hip and knee joints in the sagittal plane) toward a physiological gait control in terms of lower limb muscle rhythm and timing. Indeed, analyzing the superficial EMG measurements, we have demonstrated a selective improvement of muscular activation strategies toward the normative pattern only in the treatment group. The improvement was especially observed in the ST muscle. This muscle is primarily responsible for knee flexion and hip extension during gait (Burnfield, 2010). Before the intervention, in both groups, this muscle was generally always contracted during the swing and stance phase. After the intervention, instead, in the group treated with Ekso, we observed a regain of the controlled activation and deactivation cycle in both paretic and non-paretic legs. The ST is relaxed during the stance while it is contracting to start from the toe-off phase (Burnfield, 2010). Considering the amplitude and timing of activation, we observed a significant interaction effect for this muscle in the paretic leg, as detected by the GM and the BDSI. In particular, the BDSI index demonstrated that the ST regained the correct timing of activation (i.e., alternation of correct activation and deactivation phases) in the paretic leg. This normative pattern was, instead, not observed for the group treated with conventional care.

When coming to coherence analysis, we observed that the two groups showed a significant overall coherence shift toward a more physiological motor control in two different frequency bands of the couple of muscles ST and RF. In particular, the CG showed a significant increase in the alpha frequency band, while the experimental group, a significant increase in the gamma frequency band. A recovery in the alpha frequency band has been linked to spinal relearning, while a recovery in the beta and gamma frequency bands to a cortical relearning (Kitatani et al., 2016). We, therefore, hypothesized that the CG showed a motor control improvement in terms of functionality driven by a spinal circuits adaptation. In contrast, the experimental group shows a motor control improvement driven by cortical control-driven re-learning adaptive plasticity. In particular, considering the coherence analysis of the couple ST and RF, we focused on the terminal swing phase, where these muscles are both active. During this phase of the gait cycle, the subject is experiencing the inversion of load from one leg to the other, and this phenomenon is cortically modulated. The rhythmic motion of human lower limbs is controlled by neural circuits (i.e., central pattern generators) that produce rhythmic activation of muscles that control the limbs (Klarner and Zehr, 2018). Stroke and central nervous system lesions can damage the descending motor pathways. Walking in humans depends on the integrated action of hierarchical levels of supraspinal and spinal neural control (Kitatani et al., 2016). It has been demonstrated that intensive rehabilitative training, such as robotic-assisted training or body weight-supported treadmill training, can promote supraspinal plasticity in the motor centers involved in the locomotion (Winchester et al., 2005). When the subject is not showing the cortical modulation, the muscles are characterized by the tonic contraction, and they are always active. Indeed, in this situation, the central pattern generator is modulated only at the spinal level. When correctly working, the cortical drive performs an inhibitory action that induces the muscles relaxation and assures the control of rhythmic locomotor pattern generation and modulation (Guertin, 2013). Functional magnetic resonance imaging studies demonstrated that the carryover effect after a functional electrical stimulation-based treatment of TA was cortically mediated by the capability of the subject to correctly plan the movement at the cortical level and integrate proprioception information induced by the external movement assistance in their own control loop (Gandolla et al., 2016, 2021). We did not observe any modulation of the distal muscles in both the group of patients. TA fine control in gait is usually recovered later in the rehabilitation process since less crucial in terms of ambulation, as shown, for example, by foot drop chronicity in post-stroke subjects (Bent and Lowrey, 2013). Instead the SOL muscle has been shown to have monophasic activation in neurological patients (Gandolla et al., 2018), which may be driven by proprioceptive stimulation of the sole of the foot induced by overground walking (Bent and Lowrey, 2013; Choi et al., 2013). However, these considerations further support the hypothesis that muscular rhythm is primarily induced by fine joint support during functional movements in neurorehabilitation. In fact, Ekso motors only assist the hip and knee joints, and we observed significant changes in the muscles of the proximal compartment. In our study, the group treated with Ekso regained this modulation after the intervention. Indeed, we observed two distinct phases of activation and deactivation during the gait cycle for the ST muscle, and the coherence analysis confirmed this controlled activation. Instead, the group undergoing conventional therapy did not show this improvement, and, indeed, the ST muscle activation was not modulated. Therefore, we hypothesize that exoskeleton-mediated therapy, with its powered timed and modulated fine control at a hip and knee level, can induce a controlled and rhythmic locomotion in patients with subacute post-stroke. In fact, according to these findings, we can conclude that both groups regained the function of locomotion. However, our results could suggest that the CG was characterized by maladaptive compensatory mechanisms, while the EG showed a centrally-mediated learning adaptive physiological control mechanism. Consequently, the powered timed fine-tuning of the gait kinematics parameter during overground training through wearable powered robotic exoskeleton is essential to induce the correct relearning process.

Besides the applicability to evaluate the rehabilitative effectiveness, the EMG analysis could also be used for the step segmentation during gait analysis. Indeed, we demonstrated it to be an accurate alternative to the standard methods generally used for the kinematic analysis (e.g., footswitches, optoelectronic systems, inertial measurement units). Therefore, in the case of EMG analysis, the use of a single device could allow both muscular activation analysis along with step segmentation feature.

Despite the novelty of this study, some limitations can be identified. First, this study is not a randomized controlled trial; therefore, the absence of random allocation of patients could have influenced the obtained results, even though the statistical analysis of the baseline characteristics did not show significant differences between groups. Another important limitation is represented by the segmentation algorithm based on the EMG signal. Kinematics data, indeed, were not available and might allow step segmentation even for more impaired subjects. Finally, the number of treatments performed with Ekso was low. According to the literature, higher intensity could be more effective, and it could induce improvements also in other muscular districts.

According to the results of this study, we can hypothesize that the integration of robotic-mediated gait rehabilitation alongside standard therapy, although not showing superior performance in terms of functional outcomes, helps the plasticity rehabilitation process through a cortical-mediated relearning, which results in more rhythmic muscles activation pattern. A more physiological muscle activation pattern, in turn, may lead to a more metabolic efficient gait, possibly leading to a healthier recovery and long-term recovery.

## 5. Conclusions

This study investigated the muscular activation profiles of patients with subacute post-stroke before and after the rehabilitative process. Participants followed either standard physiotherapy alone or standard physiotherapy combined with wearable powered exoskeleton-assisted overground gait training. Both groups improved their ability to walk in terms of functional gait, as detected by standard clinical scales. However, only the group treated with the wearable powered robotic exoskeleton regained a rhythmic and controlled gait, as observed by the muscular activation patterns of proximal lower limb muscles, inducing the patient to regain a more physiological gait. Future studies should include a follow-up to determine if the improvement induced by the robotic training are maintained after the end of the intervention. Moreover, the orthotic effect of robotic devices in terms of muscular activation could be investigated. The results of this kind of analysis could be used to stratify patients and tailor their rehabilitative pathway.

## Data Availability Statement

The raw data supporting the conclusions of this article will be made available by the authors, without undue reservation.

## Ethics Statement

The studies involving human participants were reviewed and approved by Comitato Etico Interaziendale delle Province di Lecco, Como e Sondrio, Prot. 48892/15 23/11/2015. The patients/participants provided their written informed consent to participate in this study.

## Author Contributions

MG participated in the conception and coordination of the project, clinical tests, data collection, analysis, interpretation, and manuscript writing. VL participated in data analysis and interpretation, and manuscript writing. EG and FM participated in the conception of the study, the requirement definition, patient recruitment, clinical tests, data interpretation, and dealt with the clinical issues. AP participated in the conception and coordination of the project and manuscript writing. All authors discussed the results and contributed to the final manuscript and made a significant contribution to the review of the manuscript, read and approved the final manuscript.

## Funding

This study has been funded by the Slink Project.

## Conflict of Interest

AP and MG hold shares of AGADE Srl, Milano, Italy. The remaining authors declare that the research was conducted in the absence of any commercial or financial relationships that could be construed as a potential conflict of interest.

## Publisher's Note

All claims expressed in this article are solely those of the authors and do not necessarily represent those of their affiliated organizations, or those of the publisher, the editors and the reviewers. Any product that may be evaluated in this article, or claim that may be made by its manufacturer, is not guaranteed or endorsed by the publisher.
